# Novel Semiquantitative Bone Marrow Oedema Score and Fracture Score for the Magnetic Resonance Imaging Assessment of the Active Charcot Foot in Diabetes

**DOI:** 10.1155/2017/8504137

**Published:** 2017-11-05

**Authors:** L. Meacock, N. L. Petrova, Ana Donaldson, A. Isaac, A. Briody, R. Ramnarine, M. E. Edmonds, D. A. Elias

**Affiliations:** ^1^Department of Radiology, King's College Hospital NHS Foundation Trust, London, UK; ^2^Diabetic Foot Clinic, King's College Hospital NHS Foundation Trust, London, UK; ^3^Division of Diabetes and Nutritional Sciences, King's College London, London, UK

## Abstract

There are no accepted methods to grade bone marrow oedema (BMO) and fracture on magnetic resonance imaging (MRI) scans in Charcot osteoarthropathy. The aim was to devise semiquantitative BMO and fracture scores on foot and ankle MRI scans in diabetic patients with active osteoarthropathy and to assess the agreement in using these scores. Three radiologists assessed 45 scans (Siemens Avanto 1.5T, dedicated foot and ankle coil) and scored independently twenty-two bones (proximal phalanges, medial and lateral sesamoids, metatarsals, tarsals, distal tibial plafond, and medial and lateral malleoli) for BMO (0—no oedema, 1—oedema < 50% of bone volume, and 2—oedema > 50% of bone volume) and fracture (0—no fracture, 1—fracture, and 2—collapse/fragmentation). Interobserver agreement and intraobserver agreement were measured using multilevel modelling and intraclass correlation (ICC). The interobserver agreement for the total BMO and fracture scores was very good (ICC = 0.83, 95% confidence intervals (CI) 0.76, 0.91) and good (ICC = 0.62; 95% CI 0.48, 0.76), respectively. The intraobserver agreement for the total BMO and fracture scores was good (ICC = 0.78, 95% CI 0.6, 0.95) and fair to moderate (ICC = 0.44; 95% CI 0.14, 0.74), respectively. The proposed BMO and fracture scores are reliable and can be used to grade the extent of bone damage in the active Charcot foot.

## 1. Introduction

Charcot osteoarthropathy is a severe complication of diabetes. It is associated with considerable bone destruction leading to foot deformity, risk of ulceration, and sometimes amputation [1–3]. Imaging is important to confirm the diagnosis and stage of the disease, but in cases presenting early, radiographic signs may be lacking [4, 5]. However, magnetic resonance imaging (MRI) can detect early bone damage [4, 6]. Over the last 10 years, there has been considerable interest in the use of this imaging modality in the diagnosis of Charcot osteoarthropathy [3, 7]. Subchondral bone marrow oedema (BMO) and fractures are well-recognised features of the active Charcot foot, but methods to quantitate them are lacking.

The aims of this study were firstly to devise a semiquantitative BMO score and fracture score on noncontrast foot and ankle MRI scans in patients presenting with active Charcot osteoarthropathy and secondly to assess the intraobserver and interobserver agreement when using these scores.

## 2. Materials and Methods

This paper utilises imaging data from a cohort of 45 patients who took part in a double-blind randomised clinical trial with daily subcutaneous administration of recombinant human parathyroid hormone or placebo (EudraCT Number: 2009-016873-13). All observers and clinicians were blinded to the treatment allocation of the subjects. The study was carried out in a single centre over a period of 3 years. Study patients were selected from referrals to a specialist diabetic foot unit. All patients had diabetes and presented with active Charcot osteoarthropathy, as defined by a clinically acute hot swollen foot with intact skin and a skin temperature > 2°C compared with the same site on the contralateral foot (Dermatemp 1000; Exergen, Watertown, MA). The diagnosis of active Charcot osteoarthropathy was made in keeping with the diagnostic criteria defined in the recent task force document [3]. Below-knee casting was initiated in all cases at the time of clinical presentation.

Each patient underwent an MRI scan at the time of treatment randomisation (within 2 weeks of clinical presentation) prior to the initiation of therapy. All participants provided written informed consent according to the Helsinki Declaration prior to inclusion of the study, which was approved by the London-South East National Research Ethics Committee.

### 2.1. MRI Protocol

All patients underwent noncontrast MRI scan of the affected Charcot foot (Siemens Avanto 1.5T, Erlangen, Germany, dedicated foot and ankle coil was used where possible) and were examined in the supine position. Imaging parameters were as follows: axial T1 turbo spin echo (TR 471, TE 11, averages 2, slice 3 mm, slice gap 10%, and FOV 230 mm), axial STIR (TI 150, TR 3460, TE 26, averages 2, slice 3 mm, slice gap 10%, and FOV 230 mm), coronal T1 turbo spin echo (TR 527, TE 11, averages 1, slice 4 mm, slice gap 10%, and FOV 130 mm), and sagittal STIR (TI 150, TR 3240, TE 26, averages 1, slice 3 mm, slice gap 10%, and FOV 230 mm). All MRI scans were stored on the institution's picture archiving and communicating system (PACS) for further analysis.

### 2.2. MRI Scoring Proforma Development

**Figure 1:**
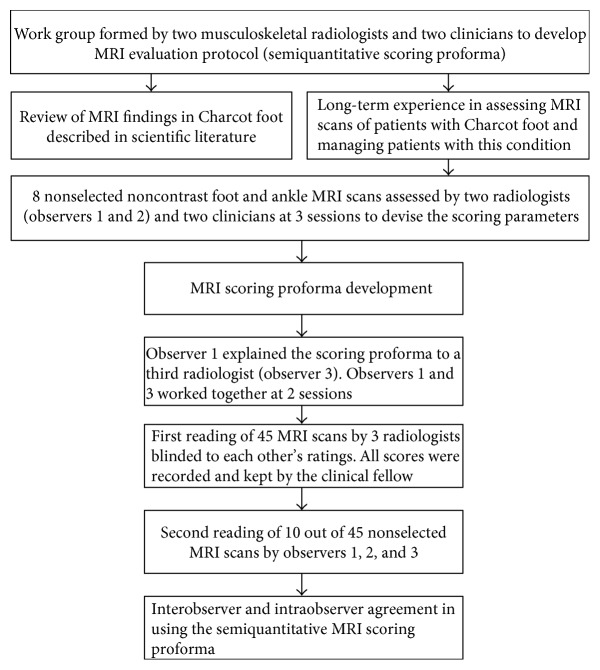
Development of MRI semiquantitative scoring proforma and reliability assessment.

A work group was formed to develop a detailed MRI evaluation protocol (semiquantitative scoring proforma) (Figure 1). This consisted of two musculoskeletal radiologists (observer 1 (DAE) and observer 2 (LM) with combined 20-year musculoskeletal radiological experience) and two clinicians (NLP and MEE, with combined 40-year clinical experience in managing patients with Charcot osteoarthropathy). The work group used GE Centricity PACS workstations with dedicated high-resolution viewing monitors.

**Figure 2:**
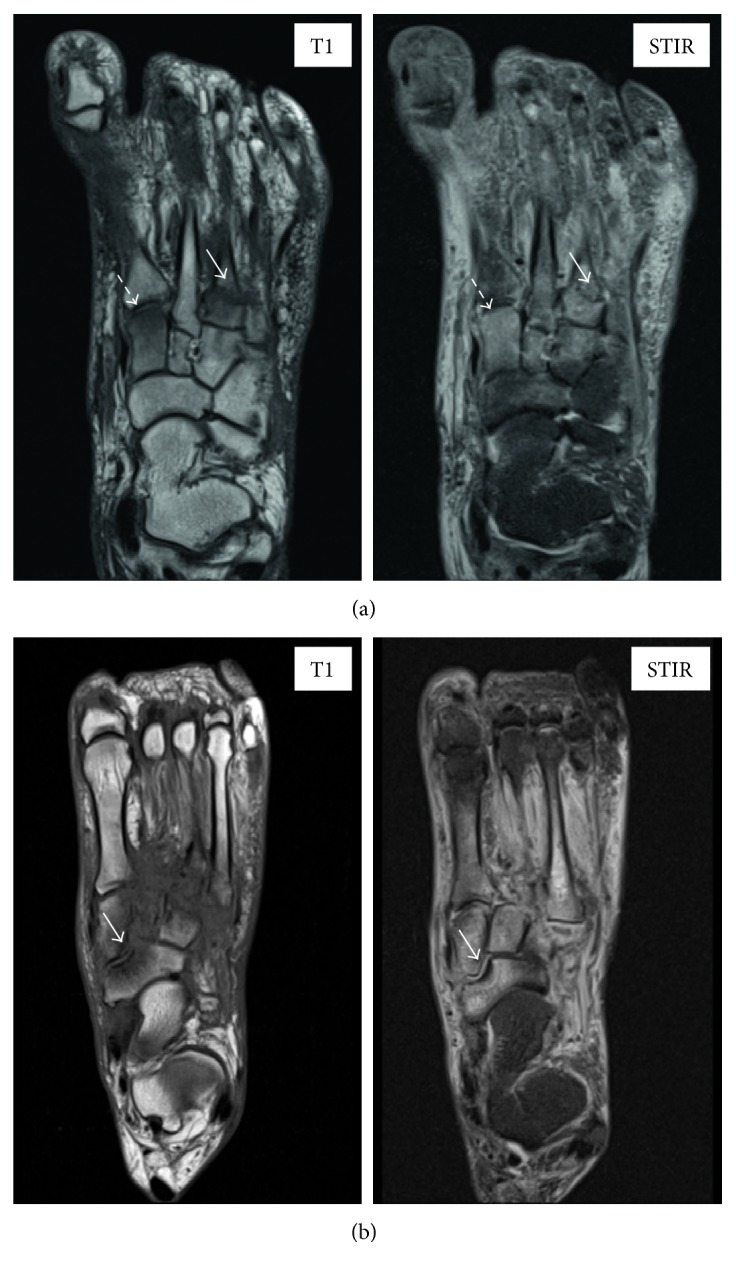
Noncontrast large field-of-view MR scans in active Charcot osteoarthropathy. Representative examples of bone marrow oedema at the medial cuneiform (BMO score = 2; white dashed arrow) and fracture at the base of the 3rd metatarsal (fracture score = 1; white arrow) noted on axial T1 and STIR MR images (a). Example of collapse of the navicular bone (fracture score = 2, white arrow) noted on axial T1 and STIR MR images (b).

Initially, 8 nonselected scans were analysed by the two radiologists (observers 1 and 2) who worked together with the clinicians to devise the scoring parameters. The radiologists and the clinicians reviewed the scans on 3 sessions. The following bones were reviewed for the presence of bone marrow oedema and fracture in all 3 imaging planes: 1st to 5th proximal phalanges; medial and lateral sesamoids; 1st to 5th metatarsals; medial, middle, and lateral cuneiforms; navicular, cuboid, talus, and calcaneum; distal tibial plafond; and medial and lateral malleoli. Bone marrow oedema (BMO) was defined as the presence of hyperintense marrow signal to normal marrow signal on STIR images, with or without corresponding abnormal hypointense marrow signal on T1-weighted imaging (Figure 2(a)). In order to minimise error from partial volume averaging, a bone was only considered to be positive for BMO if the signal was clearly located within the anatomical confines of the bone or was identified on more than one imaging plane. A fracture was defined as an abnormal low-signal line crossing a bony cortex or subchondral bone plate on T1-weighted imaging or a similar abnormal low- or high-signal line on STIR images (Figure 2(a)). Fragmentation was defined as the presence of one or more clearly separate bone fragments. Collapse was defined as an impaction deformity of an articular surface (Figure 2(b)). Each bone was scored individually for the extent of oedema (BMO score: 0—no oedema, 1—oedema < 50% of whole bone volume, and 2—oedema > 50% of whole bone volume) and for the presence of fracture (fracture score: 0—no fracture, 1—fracture, and 2—collapse/fragmentation). The anatomic patterns of involvement as defined by Sanders and Frykberg's classification were recorded as metatarsal-phalangeal joints (pattern I), metatarsal-tarsal joints (pattern II), tarsal joints (pattern III), ankle joint (pattern IV), and the posterior process of the calcaneum (pattern V) [1]. Patterns I to IV were also assessed for the presence or absence of subluxation.  Table 1 shows the developed semiquantitative proforma.

The following parameters were devised:

- Total BMO score: this comprised the sum of BMO scores of each bone. The total BMO score was calculated, and the maximum score for each scan was 44.
- Total fracture score: this comprised the sum of fracture scores of each bone, and the maximum fracture score for each scan was 44.

### 2.3. Radiological Assessment

Observers 1 and 2 reviewed all scans at individual sessions, using the devised scoring proforma. All scores were recorded by the clinical fellow (NLP) who kept all records. Subsequently, observer 1 explained the scoring proforma to a third radiologist (AI) (observer 3), a colleague with 4 years of experience in musculoskeletal radiology, who was not involved in the development of the proforma. Observers 1 and 3 worked together at 2 sessions, and subsequently, observer 3 assessed all 45 scans. All observers were blinded to each other's ratings, and all scores were recorded and kept by the clinical fellow.

To assess intraobserver agreement in reporting the total BMO score, total fracture score, patterns of involvement, and presence of subluxation, the clinical fellow randomly selected 10 out of 45 MRI scans, which were scored for a second time by each of the three radiologists individually at separate sessions, blinded to the previous scoring. The minimal interval between ratings was six months for observers 1 and 2 and one month for observer 3.

## 3. Statistical Analysis

Multilevel models were used to measure the interobserver agreement and intraobserver agreement for total BMO and fracture scores, and Bland and Altman plot analysis was used to assess bias between readings [8]. Agreement was measured using intraclass correlation coefficients (ICCs) with 95% confidence intervals (CI) for continuous variables (total BMO score and total fracture score) and Cohen's kappa coefficients with standard error (SE) for categorical variables (patterns of involvement and presence of subluxation). The benchmark limits for agreement in terms of ICCs and kappa coefficients followed established classifications [8, 9].

According to Altman's benchmark scale, ICC values of 0.81 to 1 indicated very good agreement, values of 0.61 to 0.8 good agreement, values of 0.41 to 0.6 moderate agreement, values of 0.21 to 0.4 fair agreement, and values below 0.2 poor agreement [8]. According to Landis-Koch's benchmark scale, kappa values of 0.81 to 1 showed almost perfect agreement, values of 0.61 to 0.8 substantial agreement, values of 0.41 to 0.6 moderate agreement, values of 0.21 to 0.4 fair agreement, values of 0 to 0.2 slight agreement, and values below 0 poor agreement [9]. In all cases, for more rigour, in addition to the point estimate, the lower bound of the 95% CI was taken into account [9].

## 4. Results

### 4.1. Characteristics of the Study Population

There were 35 males and 10 females, 14 had type 1 diabetes, and 31 had type 2 diabetes. The mean age and duration of diabetes was 55 years (range 27–76) and 17 years (range 1–40), respectively. The mean glycated HbA1c was 68 ± 15.3 mmol/mol (mean ± SD). The estimated glomerular filtration rate was below 60 ml/min in 10 patients.

All patients presented with an active-stage Charcot foot—eleven patients presented with grade 0 (X-ray normal and MRI abnormal) and 34 patients presented with grade 1 Charcot foot (X-ray abnormal and MRI abnormal) in agreement with the new classification based on MRI [10].

### 4.2. Interobserver Agreement

#### 4.2.1. Total BMO Score

The total BMO score ranged from 2 to 34 with mean 18.8 and standard deviation (SD) 7.1 for observer 1, from 1 to 33 with mean 17.9 and SD 7.2 for observer 2, and from 4 to 32 with mean 17.8 and SD 6.7 for observer 3.

A multilevel linear regression model indicated that there was no significant difference between the three observers (*p* = 0.21). The interobserver ICC indicated very good agreement between the three observers (ICC = 0.83, 95% CI 0.76, 0.91). The agreement was very good between observers 1 and 2 and good between observers 1 and 3 and between observers 2 and 3 (Table 2).

Pairwise differences were not statistically different. The mean difference between observers 1 and 2 was −0.83 (95% CI 2, 0.33, *p* = 0.16), that between observers 1 and 3 was −0.96 (95% CI 2.1, 0.20, *p* = 0.11), and that between observers 2 and 3 was 0.13 (95% CI 1, 1.3, *p* = 0.83).

#### 4.2.2. Total Fracture Score

The total fracture score ranged from 0 to 14 with mean 6 and SD 4.2 for observer 1, from 0 to 20 with mean 8 and SD 5.3 for observer 2, and from 0 to 22 with mean 9.5 and SD 6.4 for observer 3.

A multilevel linear regression model indicated that there was a significant difference between the three observers (*p* < 0.0001). The interobserver ICC indicated good agreement between the three observers (ICC = 0.62; 95% CI 0.48, 0.76). There was good agreement between observers 1 and 2 and observers 2 and 3, whereas the agreement between observers 1 and 3 was moderate (Table 2).

The mean difference between observers 1 and 2 was 2 (95% CI 0.85, 3.3, *p* = 0.001), that between observers 1 and 3 was 3.5 (95% CI 2.4, 4.7, *p* < 0.001), and that between observers 2 and 3 was −1.5 (95% CI 2.7, −0.3, *p* = 0.01).

#### 4.2.3. Patterns of Involvement and Presence of Subluxation

The interobserver agreement in defining zones of involvement for all observers was substantial for patterns II and III (kappa intraclass correlation coefficients were 0.68 (SE 0.14) and 0.61 (SE 0.14), resp.), moderate for pattern V (kappa intraclass correlation coefficient was 0.48 (SE 0.14)), and fair for patterns I and IV (kappa intraclass correlation coefficients were 0.39 (SE0.14) and 0.39 (SE 0.14), resp.).

The interobserver agreement in defining the presence of subluxation for all observers was moderate for subluxation of patterns I, III, and IV (kappa intraclass correlation coefficients were 0.42 (SE 0.14), 0.42 (SE 0.14), and 0.58 (SE 0.14), resp.) and fair for pattern II (kappa intraclass correlation coefficient was 0.38 (SE 0.14)).

### 4.3. Intraobserver Agreement

#### 4.3.1. Total BMO Score

The multivariate multilevel regression indicated that there was no significant difference in the total BMO score between the two readings (*p* = 0.28). The mean difference between the two readings was 0.79 (95% CI 0.64, 2.23). This was consistent for each observer (*p* = 0.41). Furthermore, Bland-Altman comparison of the two readings for the total BMO score confirmed that there was very good intraobserver agreement. It gave a reference range for difference from −9.732 to 9.375. The mean difference was −0.179 (95% CI 2.031, 1.674), indicating a nonsignificant bias between the two readings. The intraobserver ICC indicated good agreement for all observers (ICC = 0.78, 95% CI 0.6, 0.95).

#### 4.3.2. Total Fracture Score

There was a significant difference in the total fracture score between the two readings as indicated by the multivariate multilevel regression (*p* = 0.003). The mean difference between the two readings was 2.2 (95% CI 0.74, 3.6), and according to a nonsignificant interaction test, this difference was consistent for all observers (*p* = 0.36). Bland-Altman comparison of the two readings gave a reference range for difference from −9.871 to 5.309. The mean difference was −2.28 (95% CI 3.650, −0.913), significantly different from zero. However, the intraobserver ICC indicated fair-to-moderate agreement (ICC = 0.44; 95% CI 0.14, 0.74).

#### 4.3.3. Patterns of Involvement and Presence of Subluxation

The intraobserver agreement in defining zones of involvement according to Sanders and Frykberg's classification was almost perfect for pattern II (kappa intraclass correlation was 1.00) and substantial for patterns I and III (kappa intraclass correlation coefficients were 0.75 (SE 0.18) and 0.79 (SE 0.18), resp.). The agreement was moderate for patterns IV and V, and the kappa coefficients were 0.43 (SE 0.17) and 0.47 (SE 0.18), respectively. The intraobserver agreement in defining subluxation was almost perfect for patterns I and IV with kappa coefficients of 0.92 (SE 0.18) and 1.00, respectively, and very good for pattern IV with kappa coefficient of 0.75 (SE 0.18). The agreement was moderate to substantial in defining subluxation of pattern II, and the kappa coefficient was 0.61 (SE 0.16).

## 5. Discussion

This study reports the development of a detailed evaluation protocol using a semiquantitative scoring proforma on noncontrast foot and ankle MRI scans in patients with active Charcot osteoarthropathy. MRI rather than X-ray has become the modality of choice for diagnosing and monitoring this condition [7, 10, 11]. In the active stage, conventional radiographs are valuable for assessing fractures, deformity, and malalignment in grade 1 [10]. However, radiological findings are absent in grade 0 [10]. MRI is sensitive for the evaluation of skeletal pathology, and furthermore, radiographically occult fractures may be identified [4, 10]. Indeed, this imaging modality has been recommended for diagnosing Charcot osteoarthropathy in a recent international task force document [3].

Despite this widely recognised usefulness of MRI scans in the diagnosis of the Charcot foot [3, 4, 6, 7, 10], there are no accepted methods of grading bone damage. To grade BMO and fractures, which are the main pathological features of the active Charcot foot, we derived a total BMO score and a total fracture score. The proposed scoring proforma is novel and provides a structured approach to quantitate the extent of the Charcot process. Moreover, it was devised by clinicians and radiologists working together with an overall aim to translate it to everyday practice as a useful tool in the assessment of the active Charcot foot.

Although MR imaging sequences of the foot are usually performed using a small field of view dedicated to only the forefoot, midfoot, or hind foot, a large field of view was chosen to include the whole foot on all sequences. This approach inevitably limits spatial resolution, but we considered that this was outweighed by the imperative to assess the whole foot in all our patients since Charcot osteoarthropathy frequently affects multiple locations in the foot even at initial presentation. Additionally, a standard whole foot protocol for all patients would maximise the generalisability of our findings. We chose not to include post gadolinium intravenous contrast MR sequences in our study protocol, as many Charcot patients present with renal impairment and their estimated glomerular filtration rate can be below the safety range for gadolinium administration. Thus, we believe that this protocol can be safely used in every patient with a suspected Charcot foot (in the absence of absolute MRI contraindications). This approach to MR imaging sequences is appropriate in our group of patients presenting clinically with active Charcot foot, with intact skin and no clinical question of infection.

Our study demonstrated that the inter- and intraobserver agreements in reporting BMO score were better than the inter- and intraobserver agreements in reporting fracture score. This is not surprising as widespread BMO is a readily identifiable MRI feature on STIR images, whereas identifying fractures and collapse of the articular surface, particularly in the tarsal bones, can be more difficult, especially where large field-of-view images are used, and this requires experience with Charcot MRI scans. Advancing MR technology with improving gradient strengths and the increasing availability of 3 Tesla systems in clinical use will allow for improved image resolution without an increasing acquisition time, and this should improve conspicuity of fractures and increase reliability of the fracture score in future studies. Nevertheless, we feel that, despite its more limited interobserver reliability, the fracture portion of the score should be preserved in our proforma as radiographically occult fractures are a hallmark of the pathogenesis of the active Charcot foot [10, 12]. However, we recognise that critical analysis of data using the proforma should take account of the differing reliabilities of the BMO and fracture scores.

A further limitation to our study is that MRI scans were carried out 2 weeks after clinical presentation and initiation of offloading. Therefore, the extent of bone abnormalities detected on MRI may not fully reflect the initial pathological lesion or could have been affected by casting therapy. We have not discussed the pathological basis of BMO. The latter is accepted in the literature as a hallmark of the active Charcot foot, and its pathological basis, even though of interest and importance, is beyond the scope of this paper.

Clinical resolution of an acute episode of Charcot osteoarthropathy is defined by skin temperature falling below 2°C compared with the contralateral foot [13], and MR imaging studies have demonstrated a decrease in BMO, contrast enhancement, and contrast enhancement rate at clinical resolution [14]. Although resolution of BMO has sometimes been shown to be delayed relative to clinical resolution [14], we believe that our proforma could be used not only in the assessment of the active Charcot foot but also in the identification of the change in BMO scores and fracture scores in MRI scans carried out between presentation and follow-up [15].

In conclusion, we believe that this is the first study that reports a novel semiquantitative BMO score and fracture score in the assessment of the active Charcot foot. The proposed scoring proforma is reliable in the assessment of marrow oedema and in grading the extent of bone damage in the active Charcot foot. Further research is required to validate this scoring system as a clinical tool to monitor treatment and assess outcome.

## Figures and Tables

**Table 1:** Proposed King's College Hospital semiquantitative proforma for the MRI assessment of the active Charcot foot in diabetes.

Hospital number:	Surname	Name
Scan date:	Involvement (yes/no)	Subluxation (yes/no)
Pattern I		
Pattern II		
Pattern III		
Pattern IV		
Pattern V		Not applicable

Semiquantitative score	BMO score (0–2)	Fracture score (0–2)

Medial sesamoid		
Lateral sesamoid		
Proximal phalanx 1		
Proximal phalanx 2		
Proximal phalanx 3		
Proximal phalanx 4		
Proximal phalanx 5		
Metatarsal 1		
Metatarsal 2		
Metatarsal 3		
Metatarsal 4		
Metatarsal 5		
Medial cuneiform		
Intermediate cuneiform		
Lateral cuneiform		
Cuboid		
Navicular		
Talus		
Calcaneum		
Tibial plafond		
Medial malleolus		
Lateral malleolus		
Total score = sum of all scores		

**Table 2:** Interobserver agreement for the total BMO score and total fracture score for all observers and for the three pairs of observers.

	Total BMO score ICC (95% CI)	Total fracture score ICC (95% CI)
All observers	0.83 (0.76, 0.91)	0.62 (0.48, 0.76)
Observer 2 versus observer 1	0.93 (0.88, 0.97)	0.66 (0.50, 0.82)
Observer 3 versus observer 1	0.77 (0.64, 0.89)	0.49 (0.27, 0.70)
Observer 3 versus observer 2	0.80 (0.69, 0.91)	0.70 (0.56, 0.85)
